# Acute cervical radiculopathy after anterior scalene muscle massage: A case report

**DOI:** 10.1097/MD.0000000000033560

**Published:** 2023-04-14

**Authors:** Sung Joon Chung, Yunsoo Soh

**Affiliations:** a Department of Physical Medicine and Rehabilitation, Kyung Hee University Hospital at Gangdong, Seoul, Republic of Korea; b Department of Physical Medicine and Rehabilitation Medicine, Kyung Hee University Hospital, Seoul, Republic of Korea.

**Keywords:** anterior scalene muscle, cervical nerve root, massage, radiculopathy

## Abstract

Deep tissue massage (DTM) is a form of therapeutic massage therapy for muscles and is often used to treat musculoskeletal pain. This was an uncommon case of acute cervical radiculopathy that occurred after DTM.

**Patient concerns:**

A 47-year-old Asian woman with low weight visited our clinic due to complaints of sudden unilateral paralysis, radiating pain in the left shoulder, and wrist weakness after undergoing a 3-minute DTM of the anterior scalene muscle. Electrodiagnostic examination indicated acute injuries in the left cervical 5 and 6 (cervical 5 and cervical 6) nerve roots.

**Diagnoses:**

Acute cervical radiculopathy associated with anterior scalene DTM.

**Intervention:**

The patient underwent ultrasound-guided cervical 5 and cervical 6 selective nerve root block twice through the injection of 0.25% lidocaine and 20 mg dexamethasone and regularly participated in a biweekly rehabilitation program and a home exercise program.

**Outcome:**

After a 6-month follow-up, the patient’s shoulder and wrist strength had recovered, and the electrodiagnostic findings had improved.

**Lessons:**

DTM of the anterior scalene muscle should be carefully performed to avoid cervical nerve root injury, particularly in underweight patients.

## 1. Introduction

Cervical radiculopathy is a neurological condition caused by nerve root compression injury and inflammatory reactions around the nerve root or foramen.^[1]^ The common symptoms and signs of cervical radiculopathy are radiating upper limb pain, paresthesia or numbness, muscle weakness, and pain in the posterior neck and scapular area.^[1]^ The most common causes are cervical disc herniation and cervical spondylosis.^[1]^ Deep tissue massage (DTM) is a form of therapeutic massage therapy for muscles and is often used for the treatment of musculoskeletal pain in order to relax the tensed muscles.^[2]^ Herein, we report an uncommon case of acute cervical radiculopathy after DTM of the anterior scalene muscle (ASM).

## 2. Case presentation

A 47-year-old Asian woman visited our clinic due to complaints of radiating pain and weakness in the left shoulder, elbow, and wrist. She had no past history of chronic diseases or medication use; she was 154 cm tall and weighed 41 kg. The patient’s body mass index was 17.2, which was classified as underweight according to the World Health Organization Asian weight standards.^[3]^ The patient experienced contralateral right side shoulder pain 1 month prior to admission. The patient visited the musculoskeletal pain clinic, was diagnosed with myofascial pain syndrome, and underwent bilateral scalene muscle massage. After a 3-minute massage in the left ASM, sudden radiating pain and developed shoulder, elbow, and wrist weakness occurred.

Manual muscle test was performed and revealed grade 2 weakness during left shoulder abduction and forward flexion of elbow flexors, and grade 3 weakness of wrist extensors (2, active movement with gravity eliminated; 3, active full movement against gravity, but not resistance); other motor grades showed absence of weakness. She presented with radiating pain and paresthesia along the cervical 5 and 6 [cervical 5 (C5) and cervical 6 (C6)] dermatomes. The active and passive ranges of motion (ROMs) of the neck and left shoulder joints were normal. Spurling tests for cervical root signs showed negative results. The deep tendon reflexes (DTRs) of the left biceps brachii (main C5) and brachioradialis (main C6) tendons showed hyporeflexia, while the other tendon reflexes were normal. There was no evidence of upper neuron signs, such as the Babinski and Hoffman sign, cranial nerve dysfunction, dysarthria, or incontinence. Hence, cervical spine magnetic resonance imaging (MRI) was performed and showed a right-sided C5–6 disc with mild subarticular protrusion with absence of nerve compression. Definite lesion on the left side disc herniation or central nervous system injury, including the cervical spinal cord, was not observed. Damage to the cervical nerve root of the peripheral nervous system was suspected, considering the radiating upper limb pain, hyporeflexia of DTRs, weakness, and sensory deterioration. Under the diagnostic impression of acute cervical nerve injury to the C5 and C6 nerve roots, an ultrasound-guided C5 and C6 selective nerve root block was performed by injecting 0.25% lidocaine and 20 mg dexamethasone.

Four weeks after symptom onset, nerve conduction studies (NCS) and electromyography (EMG) were performed. On needle EMG, the left C5 and C6 innervated muscles, including the cervical paraspinal muscle, flexor carpi radialis, extensor carpi radials, biceps, deltoid, and infraspinatus, showed recent denervation, increased fibrillation potentials, positive sharp waves, reduced recruitment, and an interference pattern (Table 1). The motor NCS showed a decreased (<40%) amplitude at the left axillary nerve (deltoid recording) and left musculocutaneous nerve (biceps recording) compared with that at the right side. Meanwhile, the motor and sensory NCS results and F responses of the median, ulnar, and radial nerves were normal. The sensory NCS of the musculocutaneous nerve (lateral and medial antebrachial) also showed normal results (Table 1). As the NCS and EMG findings showed denervation of the left C5 and C6 root levels, the patient was diagnosed with acute left C5 and C6 radiculopathy due to nerve root ischemic injury. Moreover, physical examination showed motor weakness of the left shoulder, elbow flexor, and wrist extensor; decreased tendon reflexes in the biceps and brachioradialis muscles; and sensory decline along the left C5 and C6 dermatomes, which were consistent with the electrodiagnostic results.

**Table 1:** Baseline nerve conduction study and needle electromyography.

Nerve	Stimulus site	Recording site						
Motor (mV)			Latency (ms)		Amplitude (mV)		Velocity (m/s)	
Left median	Wrist	APB	2.4		14.8			
	Elbow	APB	5.2		14.9		62.5	
Left ulnar	Wrist	ADM	2.35		13.8			
	Below Elbow	ADM	5.2		13.7		64.9	
Left axillary	EP	Deltoid	3		17.2			
Left musculocutaneous	EP	Biceps	3.5		16.9			
Sensory (μV)								
Left median	Wrist	3rd finger	2.7		9.3		51.9	
Left ulnar	Wrist	5th finger	2.85		8.1		49.1	
Left radial	Forearm	Snuffbox	2.5		5.3		56	
Electromyography								
Spontaneous				Voluntary MUAP				
Muscle	IA	Fibs	PSWs	Fasciculation	Amplitude	Duration	PP	Recruitment
LT. FCR	↑	3+	1+	0	N	N	N	Reduced
LT. ECR	↑	0	2+	0	N	N	N	N
LT. BB	↑	0	2+	0	N	N	N	N
LT. DELTOID	↑	0	1+	0	N	N	N	Reduced
LT. IS	↑	2+	2+	0	N	N	N	Discrete
LT. SA	N	0	0	0	N	N	N	N
L. C5 PS	N	1+	0	0	1+	N	N	Discrete
L. C6 PS	N	1+	0	0	1+	N	N	Reduced

All sensory and mixed latencies are regarded as peak latencies. All sensory and mixed nerve conduction velocities were calculated using onset latencies.

ADM = abductor digiti minimi, APB = abductor pollicis brevis, BB = biceps brachii, C5 = cervical 5, C6 = cervical 6, ECR = extensor carpi radialis, EP = Erb’s point, FCR = flexor carpiradialis, Fibs = fibrillation potentials, IA = insertional activity, IS = infraspinatus, LT = left, PS = paraspinalis, PSWs = positive sharp waves, SA = serratus anterior.

The patient underwent a rehabilitation program twice a week to improve the ROM in the left shoulder, elbow, and wrist. Four weeks after the first ultrasound-guided selective nerve injection, radiating pain improved by 50% and weakness gradually recovered. Hence, the patient underwent another ultrasound-guided selective nerve injection. Six months after onset, the VAS pain scores significantly improved, and the left upper extremity reflexes returned to normal. Moreover, the manual muscle test results (grade 5) and DTR in the biceps and brachioradialis muscles were normal. Follow-up NCS and needle EMG at 6 months showed reduced recruitment and an interference pattern without abnormal denervation in the cervical paraspinal muscle, flexor carpi radialis, and infraspinatus, therefore indicating progressive reinnervation after denervation (Table 2).

**Table 2:** Results of electromyography studies at 6-month follow-up.

Electromyography								
Spontaneous				Voluntary MUAP				
Muscle	IA	Fibs	PSWs	Fasciculation	Amplitude	Duration	PP	Recruitment
L. FCR	↑	0	0	0	N	N	N	Reduced
L. ECR	↑	0	0	0	N	N	N	N
L. BB	↑	0	0	0	N	N	N	N
L. DELTOID	↑	0	0	0	N	N	N	N
L. IS	↑	0	0	0	N	N	N	Reduced
L. SA	N	0	0	0	N	N	N	N
L. C5 PS	N	0	0	0	N	N	N	Reduced
L. C6 PS	N	0	0	0	N	N	N	Reduced

ADM = abductor digiti minimi, APB = abductor pollicis brevis, BB = biceps brachii, C5 = cervical 5, C6 = cervical 6, ECR = extensor carpi radialis, EP = Erb’s point, FCR = flexor carpiradialis, Fibs = fibrillation potentials, IA = insertional activity, IS = infraspinatus, LT = left, PS = paraspinalis, PSWs = positive sharp waves, SA = serratus anterior.

## 3. Discussion

**Figure 1.**
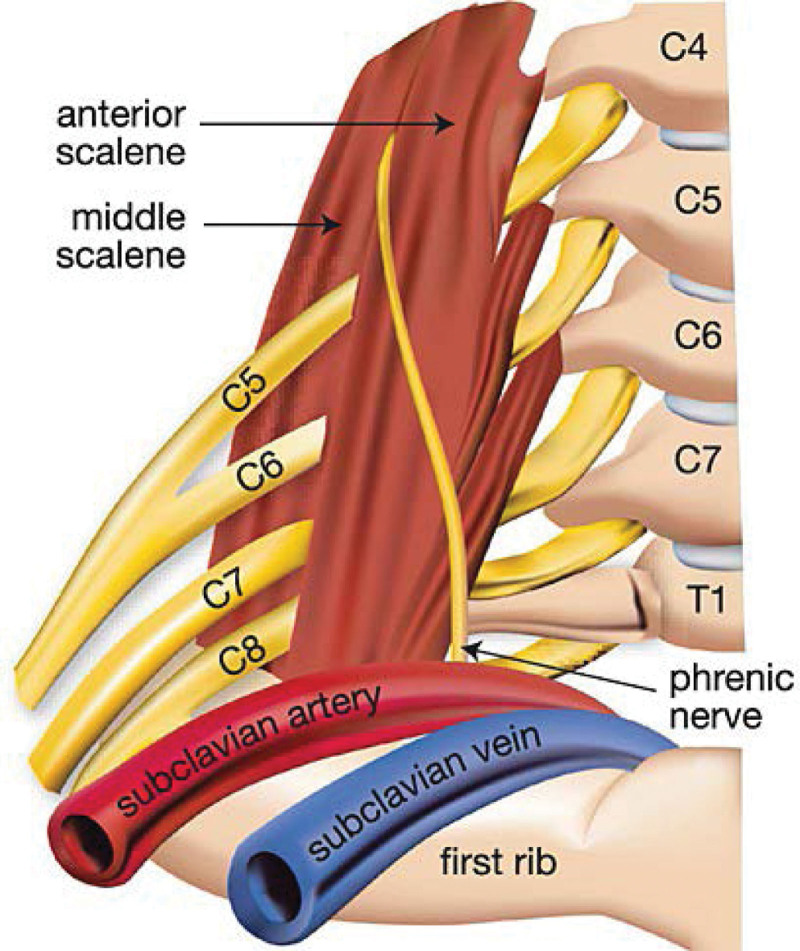
The usual pathway of the C5 and C6 nerve roots passing behind the anterior scalene muscle. Adapted from Flavel et al Sonography 2014;1(1):25–29, with permission.^[5]^ C5 = cervical 5, C6 = cervical 6.

Herein, we report a case of acute cervical radiculopathy caused by ASM massage. To the best of our knowledge, only a few previous case reports of peripheral nerve damage caused by massage therapy have been conducted. However, this is the first case report on cervical root nerve injury. The patient was a underweight Asian woman with a body mass index of 17.2. As her skin and muscles were relatively thin, she had less protection and was vulnerable to compression injury. The ASM originates from the anterior tubercles of the third to 6th transverse processes of the cervical vertebrae and is inserted into the upper face of the first rib. The C5 and C6 run between the anterior and middle scalene muscle^[4]^ (Fig. 1). The transverse process and articular facet joint of the cervical spine are located behind the C5 and C6 nerve roots; therefore, it is relatively immobile and non-distensible owing to its firm structure.^[4]^ When the physical therapist performed the ASM massage therapy, the cervical C5 and C6 nerve roots were possibly compressed, thus causing injury.

Several studies have examined the association between entrapment C5 nerve root pain symptoms in the ASM.^[5]^ When the C5 nerve root crosses the ASM due to anatomical variations, symptoms such as C5 radicular pain occur due to MPS of the ASM. In our case, the patient initially complained of contralateral right shoulder pain. However, cervical-MRI showed the absence of lesion compressing the left side nerve root. NCS and needle EMG confirmed the presence of C5 and C6 nerve root injury. Therefore, compression trauma due to deep tissue massage rather than radicular pain due to MPS of the ASM or cervical disc herniation was considered.

The prognosis of nerve damage caused by deep tissue massages remains unclear as previous case reports showed variations in age, sex, and location of injury. Chang et al^[6]^ reported upper trunk brachial plexus injury after shoulder girdle massage therapy in 58-year-old Asian women. The patient underwent DTM for MPS in the nuchal and shoulder areas. Gradual improvement was observed at 12 months after rehabilitation treatment. Giese et al^[7]^ reported posterior interosseous syndrome after DTM of the forearm in a 45-year-old male patient. The patient’s symptoms improved after 3 weeks of steroid and splint treatment. However, the nerve damage caused by DTM is not completely restored in some patients. Aksoy et al^[8]^ reported upper trapezius paralysis leading to scapular winging due to spinal accessory nerve injury caused by DTM of the shoulder to relieve the MPS in a 38-year-old female patient. ROM exercises were performed for 2 years. Hence, the patient’s function was completely restored, and the pain was relieved.

In this study, the presence of direct nerve root injury was not confirmed as brachial plexus MRI was not performed. Brachial plexus MRI can be used to confirm the diagnosis in addition to an electrodiagnostic study.

## 4. Conclusion

In our patient, the motor NCS showed decreased amplitude in the axillary and musculocutaneous nerves with axonal loss; however, the NCS showed improvement at 6 months. As the patient received steroid injections and underwent physical therapy, it was impossible to compare the effects of these 2 interventions. However, since the patient’s radiating pain symptoms showed significant improvement immediately after the local steroid and anesthetic injection, the treatment was considered effective.

Herein, we report a case of acute cervical C5 and C6 radiculopathy after DTM. As direct nerve root compression may occur, caution is required during ASM, especially when performed in underweight women.

## Acknowledgements

This manuscript acquired the editorial certificate from “Editage” by Cactus (https://online.editage.co.kr/).

## Author contributions

**Conceptualization:** Sung Joon Chung, Yunsoo Soh.

**Investigation:** Yunsoo Soh.

**Methodology:** Yunsoo Soh.

**Supervision:** Yunsoo Soh.

**Validation:** Yunsoo Soh.

**Writing – original draft:** Sung Joon Chung, Yunsoo Soh.

**Writing – review & editing:** Sung Joon Chung, Yunsoo Soh.
